# The Ascent of the Abundant: How Mutational Networks Constrain Evolution

**DOI:** 10.1371/journal.pcbi.1000110

**Published:** 2008-07-18

**Authors:** Matthew C. Cowperthwaite, Evan P. Economo, William R. Harcombe, Eric L. Miller, Lauren Ancel Meyers

**Affiliations:** 1 Institute for Cellular and Molecular Biology, University of Texas at Austin, Austin, Texas, United States of America; 2 Section for Integrative Biology, University of Texas at Austin, Austin, Texas, United States of America; University of California San Diego, United States of America

## Abstract

Evolution by natural selection is fundamentally shaped by the fitness landscapes in which it occurs. Yet fitness landscapes are vast and complex, and thus we know relatively little about the long-range constraints they impose on evolutionary dynamics. Here, we exhaustively survey the structural landscapes of RNA molecules of lengths 12 to 18 nucleotides, and develop a network model to describe the relationship between sequence and structure. We find that phenotype abundance—the number of genotypes producing a particular phenotype—varies in a predictable manner and critically influences evolutionary dynamics. A study of naturally occurring functional RNA molecules using a new structural statistic suggests that these molecules are biased toward abundant phenotypes. This supports an “ascent of the abundant” hypothesis, in which evolution yields abundant phenotypes even when they are not the most fit.

## Author Summary

Evolutionary biology tells us much about the immediate fate of a mutation once it appears, but relatively little about its long-term evolutionary implications. Major evolutionary transitions from one trait to another may depend on a long sequence of interacting mutations, each arising by chance and surviving natural selection. In this study, we characterize the network of mutations that connect diverse molecular structures, and find that this network biases evolution toward traits that are readily produced by one or a short sequence of mutations. This bias may prevent the evolution of optimal traits, a phenomenon they call the “ascent of the abundant.”

## Introduction

Despite its familiar slogan—“survival of the fittest”— evolution by natural selection may not always yield optimal organisms. In particular, it will be fundamentally constrained by the variation introduced into populations by mutation or migration. If better traits never arise, then natural selection will never have the opportunity to favor them. Whereas adaptive constraints are central to evolutionary theory [1]–[3], there have been relatively few empirical characterizations of them [4]–[8]. Several of these studies suggest that selection can overcome putative constraints [6]–[7]. Yet, one study of the enzyme beta-isopropylmalate dehydrogenase (IMDH) concludes that adaptation is constrained by its spectrum of mutations [8].

With the introduction of the fitness landscape metaphor, Sewell Wright was one of the first to argue for the importance of adaptive constraints [9]. In contrast to Fisher's panselectionist views [10], Wright suggested that fitness valleys—low-fitness genotypes separating high-fitness genotypes—may preclude simple incremental evolution [9]. He argued that adaptation depends on both the structure of the fitness landscape (that is, the spectrum of possible mutations) and demographic conditions. Since the 1930s, the theory of evolutionary constraints has matured, but is largely premised on hypothetical fitness landscapes or very local estimates of mutational effects [11],[12].

For most phenotypes of interest, we cannot yet model complete fitness landscapes. It requires knowing the fitnesses across large sets of genotypes, typically too vast to exhaustively study either empirically or computationally. There are, however, a few biologically important phenotypes for which this is tractable. In particular, Eigen and Schuster pioneered the study of RNA molecules, using RNA secondary-structure folding algorithms as tractable genotype-to-phenotype maps [12],[13]. In their model, the genotype of a molecule is its primary sequence and the phenotype is its predicted minimum free energy secondary structure; fitness is based entirely on the similarity of a phenotype to an ideal target structure. Through extensive sampling (that is, folding many diverse sequences) and evolutionary simulations, this system has motivated and clarified several important ideas in modern evolutionary theory, including error catastrophes, quasispecies, neutral networks, and punctuated equilibria [14]–[23].

The most influential concept to emerge from these RNA studies is that of “neutral networks”, which are sets of genotypes with identical fitness that are interconnected by neutral mutations [15]. In the RNA model, the genotypes in a neutral network are sequences that fold into the same shape and are connected to each other by paths of neutral point mutations. The neutral networks of RNA and protein molecules appear to share three basic characteristics: (i) most neutral networks are small (contain few genotypes), whereas relatively few are large (contain many genotypes); (ii) large neutral networks are mutationally adjacent to a greater diversity of phenotypes than small neutral networks; and (iii) large neutral networks span the entire sequence space [15], [24]–[26].

Based on these characteristics, researchers have proposed that large neutral networks should facilitate evolution by allowing populations to explore vast regions of regions of fitness landscapes through neutral drift [15],[18],[24],[26],[27]. There is some evidence to support this assertion, though it is largely based on sampling studies [15],[24],[26] or simulation studies with strong assumptions about the fitness landscape [26]. Most recently, Wagner (2008) showed that populations evolving on large neutral networks sample more alternative phenotypes than those evolving on small neutral networks, yet these populations were constrained to explore a single neutral network.

Whether large neutral networks actually facilitate the evolution of optimal phenotypes fundamentally depends on the global structure of mutational connections between different neutral networks. If large neutral networks are almost exclusively connected to other large neutral networks, then populations will easily move among common phenotypes, but be unable to evolve rare phenotypes. Theoretical and computational characterizations of RNA fitness landscapes suggest that this may, in fact, be the case. Yet, these predictions are largely based on relatively small samples of sequences which may include only the most common phenotypes in the fitness landscape [15],[24].

Here, we use the RNA folding model to determine the complete structure of fitness landscapes and how neutral network size and adjacencies constrain evolutionary dynamics (for better or for worse). Specifically, we fold all RNA molecules of lengths 12 to 18 nucleotides, and then develop a network model describing the patterns of mutational connectivity among the phenotypes produced by molecules of the same length. We build on previous characterizations of RNA neutral network structure [15],[25],[28],[29], and argue that the mutational connectivity among phenotypes follows simple predictable patterns that fundamentally constrain evolution.

## Materials and Methods

### RNA Folding Model

RNA molecules fold into secondary structures that are the essential scaffolds for functional tertiary structures and are evolutionarily conserved for most functional RNA molecules [30]. The formation of secondary structures is relatively well understood and can be rapidly predicted using thermodynamic minimization [31]–[34]. We used the Vienna RNA folding software [version 1.6.1 with the default parameter set; [33] to predict the lowest free energy shapes of all RNA molecules of lengths 12–18 nucleotides. We assume that the shape of a molecule is a reasonable proxy for its fitness [19],[21],[23] and refer to each map from sequences of length *n* to their predicted shapes as an *n*-mer fitness landscape.

### Simulation Model

We studied evolutionary dynamics on the 12-mer fitness landscape by computationally simulating a population of evolving RNA molecules. The molecules stochastically replicate at each discrete generation in proportion to their fitnesses, and evolve by point mutations. We and others have used similar models to study many aspects of RNA evolutionary dynamics [18]–[23],[35]. An important feature of the RNA system is that the fitness effect of a point mutation stems from a biologically explicit model of molecular structure and is not simply selected from a probability distribution of mutational effects, as in simpler evolutionary models.

To compute the fitness of a molecule, we first predict its minimum free energy secondary structure (that is, its groundstate), and then compare this predicted structure with a pre-specified target structure. Specifically, if *σ* is the groundstate of a molecule *m* and *t* is the target structure, then the fitness of the molecule *W* is given by
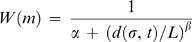
(1)where *α* = 0.01 and *β* = 1 are scaling constants, *d*(*σ*,*t*) is the Hamming distance between the parenthetical representations of *σ* and *t*, (parenthetical notation represents paired bases with pairs of parentheses and unpaired bases with dots (e.g., (((....))) is a simple stem-loop structure) and *L* = 12 is the length of the sequence. The range of fitness values possible given our choice of parameters is 0.99 - 100.0; except the open-chain shape, which was assigned a fitness of zero. Several other studies using this computational model have shown that the qualitative results are largely insensitive to the choice of parameters and even the shape of the fitness function [18]–[21],[23].

For every starting structure-target structure combination, we adapted 20 replicate populations for *τ* = 1,000,000 generations. The population size was held fixed at *N* = 1000, which was chosen both for computational tractability and to limit the effects of genetic drift. The genomic mutation rate was maintained at *U* = 0.0003 (*NU* = 0.3) for all bases in the RNA alphabet. We used soft-selection (constant *N*) to maintain the population size when genotypes that fold into the open-chain shape occasionally appear.

The expansive and intertwining neutral networks smooth the fitness landscape so that virtually every phenotype can mutate to at least one fitter phenotype, except, of course, the optimal (target) phenotype. Yet the likelihood of finding a more fit mutation while drifting on a large neutral network may be exceedingly small. Specifically, 96.7% of all neutral networks have at least one beneficial mutation (across all fitness functions considered in this study), and there always exists a path of beneficial and neutral mutations leading to the target phenotype.

In our simulations, the average time to target was 339111.7 generations; and there is no significant correlation between time to target and the abundance of the target. The simulations were allowed to run for approximately three times longer than the typical time to acquire the target, and 100 times longer than the evolutionary simulations reported in other studies using this system [18]–[21],[23]. Two sets of simulations with different parameter sets (*N = *500, *U = *0.05, *τ* = 5,000; *N* = 1000, *U* = 0.005, *τ* = 250,000) produced similar results to those reported here (not shown). The parameters were selected to be biologically reasonable and do not appear to strongly affect the outcome. Although even the most unlikely phenotype can evolve given infinite time, we believe that our results reflect the likely course of evolution.

### Rfam Informatics Analysis

Rfam is a curated database of functional RNA genes, which are those genes in which the RNA molecule itself takes parts in a biological reaction [36]. Here, we used version 7 (2006) of the database. We restricted our analysis to families in which the predicted shape of each sequence in the family was at least 60% identical to the consensus structure, thereby minimizing the effects of folding inaccuracies. This included 239 Rfam families (about 50% of the entire database) with representatives of every functional class in the database.

**Figure 1:**
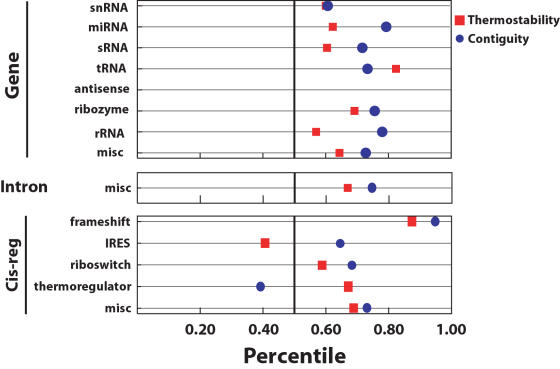
Contiguity statistic and thermostability percentiles for natural functional molecules from the Rfam database. The blue circles represent percentiles calculated from consensus structures and individual sequences, respectively. The red squares represent percentiles for thermostability predictions of molecules folding into the wildtype structures. We used 239 families in which the consensus structure was relatively well conserved among the individual genotypes. The x-axis gives the fraction of random phenotypes that are predicted to be less abundant (or less thermostable) than the actual phenotype, based on a comparison to 500 randomized molecules. The functional taxonomy is determined by the Rfam database.

Abundance estimates were obtained by calculating contiguity statistics for the secondary structures predicted by thermodynamic minimization of each sequence in a family. We then determined the rank percentiles of these abundance estimates in a null distribution of abundance estimates from random sequences. To generate the null distributions, we randomized each sequence in a family 500 times (preserving nucleotide composition), and then calculated the contiguity statistics of the ground-state shapes of these random molecules. We finally determined the fraction of contiguity statistics in the null distributions that were less than the contiguity statistic from the naturally occurring molecule (Figure 1).

### Receiver Operating Curves

Receiver operating curve (ROC) analysis is a technique for assessing the performance of classifier models [37]. The area under an ROC gives the probability that a model correctly assigns a binary variable (in this case, natural or random molecule) to its proper group. We used ROC analysis to assess relative accuracies of thermostability and contiguity for classifying sequences as natural (taken from the Rfam database) or random, under the assumption that natural molecules will have higher contiguity and thermostability than random permutations of those molecules.

Specifically, we performed logistic regressions of molecule class (natural or random permutation) on contiguity statistic and thermostability, and compute the area (*A*) under the ROC as:
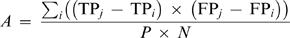
where *P* and *N* are the numbers of positive and negative instances in the data set, TP and FP are the counts of true positive and false positive classifications between indices *i* and *j*. We used the ROCR package to perform all such calculations in R 2.5.0 [38].

## Results

### Characteristics of RNA Fitness Landscapes

We have predicted the groundstate structures of all RNA molecules of lengths 12 through 18 nucleotides; we refer to length *n* RNA molecules as *n*-mers. The map from sequences to shapes is extremely degenerate with large numbers of sequences (genotypes) giving rise to identical shapes (phenotypes), as previously observed [15]–[17]. We found that the number of unique phenotypes approximately doubles with each single-base addition, from 59 unique 12-mer shapes to 3211 unique 18-mer shapes. Some of these shapes are quite common, with many unique genotypes folding into them, while others are quite rare, formed by few unique genotypes.

**Figure 2:**
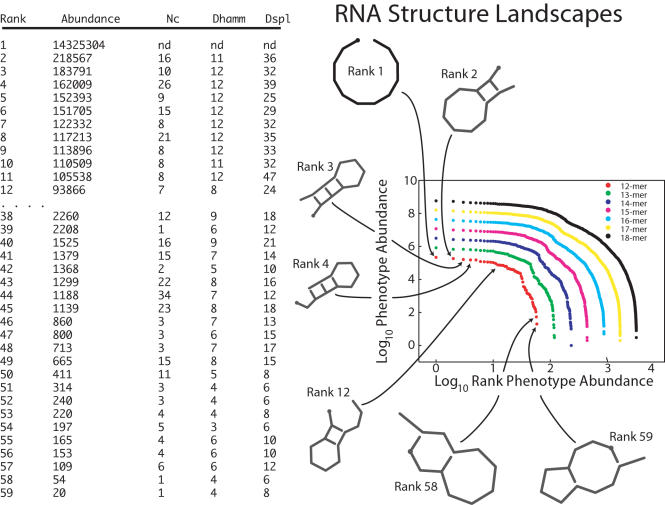
Phenotype abundance distributions for all fitness landscapes. The graph shows the phenotype abundances (y-axis) for each phenotype, ranked in order of abundance (x-axis). The most common phenotype is rank 1, the second most common is rank 2, and so on. Also shown is the distribution of abundances for the 12-mer RNA landscape (at left), along with some representative structures from this landscape.

We define *abundance* as the number of genotypes that produce a particular phenotype. The distributions of phenotype abundances appear similar across all lengths of molecules (roughly exponential without the 10% of extreme values in each tail), with relatively few highly abundant phenotypes and many rare ones Figure 2. This is qualitatively similar to the distributions reported previously for both protein and larger RNA molecules [15]–[17],[29].

Figure 2 shows a portion of the abundance distribution and a sample of shapes present in the 12-mer fitness landscape. For the 12-mer to 16-mer sequence lengths, the landscapes are composed entirely of variations on stem-loop-structures. In the 17- and 18-mer landscapes, we observe the emergence of sequences folding into multi-loop shapes, albeit at very low frequencies (on the order of 0.001% of all sequences). The relatively low structural diversity is consistent with known constraints on RNA structural motifs, for example, loops must contain at least three nucleotides [31],[33].

**Figure 3:**
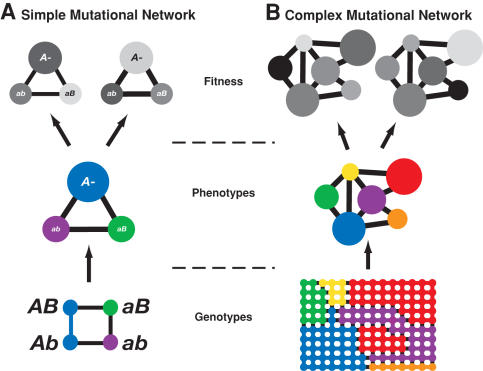
Simple mutational networks. (A) a two-locus, two-allele network and (B) a more complex (hypothetical) mutational network. The lower networks show mutational connections among genotypes; vertices are unique genotypes and edges are point mutations. Colored edges represent neutral mutations, which connect genotypes with the same phenotype (color); black edges represent non-neutral mutations, which lead to a change in phenotype. The middle networks show mutational connections among phenotypes. The size of a phenotype vertex is proportional to the number of genotypes that produce it. Pairs of vertices are connected if there is at least one point mutation that converts one phenotype to the other. The top networks show possible fitness landscapes in which each phenotype is assigned a fitness value, indicated in grayscale.

A set of genotypes that shares a common phenotype is called the *neutral network* of that phenotype (Figure 3) [15]. Neutral networks may be composed of one or more components. Within any component, all genotypes are connected to each other by a sequence of point mutations that remain within the component; these mutations are, by definition, neutral. For example, in the bottom network of Figure 3B, the red phenotype has a neutral network with two components, each of which consists of a set of red nodes interconnected by red edges. The abundance of a phenotype is precisely the size of its neutral network.

Counterintuitively, there is only a weak positive relationship between the abundance of a phenotype and the number of distinct components in its neutral network (*r* ^2^ = 0.11, *P*≈0.01). The majority of the 12-mer RNA neutral networks are dominated by relatively few large components, which each contain approximately 8–10% of the sequences in the neutral network; together these large components account for at least 80% of the neutral network. Importantly, the large components share many of the same characteristics as the entire neutral network. In particular, they are each mutationally connected to the majority of the shapes that are adjacent to the entire neutral network (typically >75%). Figure 2 also reports the number of components (Nc), the maximum Hamming distance between a pair of sequences in a single component (Dmax), and the maximum shortest path length between a pair of sequences in a single component (Dspl) for the neutral networks in the 12-mer landscape. The neutral networks for the most abundant phenotypes percolate through the entire space of genotypes.

### Characteristics of RNA Mutational Networks

The various phenotypes within a fitness landscape are connected to each other by mutations. If we aggregate all genotypes into their respective neutral networks, we create a *mutational network* in which each vertex represents a distinct phenotype and edges connect pairs of vertices when there is at least one point mutation that converts one phenotype to the other (Figure 3). For example, consider a two-locus, two-allele, haploid model with genotypes *AB*, *Ab*, *aB*, and *ab* (Figure 3A). There are three unique phenotypes–the two (*A-*) genotypes produce one phenotype (blue), *aB* produces another phenotype (green), and *ab* produces a third phenotype (purple). Mutational networks, in turn, form the underpinnings for fitness landscapes, which depend on the map from phenotype to fitness. Figure 3B caricatures a higher dimensional genotype network and its projections to phenotype and fitness networks. For RNA molecules, the vertices in a mutational network represent unique shapes and the edges represent point mutations that cause a molecule to fold into a new shape.

Roughly speaking, evolution by natural selection moves populations along the edges in a mutational network from one phenotype vertex to another. We are therefore interested in how the structure of mutational networks influences evolutionary dynamics. Intuitively, the structure of a mutational network may influence (i) the likelihood that a given phenotype will arise and, (ii) if it arises, the likelihood that the population can further evolve other, better phenotypes. Hereafter, we use *accessibility* to refer to the likelihood that a phenotype will arise, and *evolvability* as the likelihood that a phenotype can further evolve other, better phenotypes.

The most straightforward measure of a phenotype's mutational connectivity is its *degree* in the mutational network, that is, the number of other phenotype that can be reached by a single mutation. For the 12-mer through 18-mer RNA molecules, there are significant positive correlations between phenotype abundance and degree [*R* = 0.88 (12-mer) to *R* = 0.91 (18-mer); *P*<2×10^−16^]. This has been observed previously and suggests that abundant phenotypes should be both more evolvable and more accessible than rare phenotypes [24],[26],[27].

The degree of a phenotype is, however, a crude indicator of its mutational connectivity to other phenotypes. It does not reflect the probability that a mutation will actually yield a new phenotype; this probability typically declines as the size of the neutral network increases. Furthermore, the degree does not quantify whether the non-neutral mutations off a neutral network are evenly divided among the set alternative phenotypes, or are biased towards a select few of these phenotypes.

We therefore developed two novel statistics, which provide a more nuanced perspective on mutational connectivity. Both of these statistics use the quantity 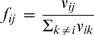, where *ν_ij_* is the number of point mutations to genotypes in the neutral network for phenotype *i* that create a genotype in the neutral network for phenotype *j*, and 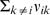 is the total number of non-neutral point mutations to genotypes in the neutral network for phenotype *i*. Thus, *f_ij_* is the fraction of non-neutral point mutations to genotypes in the neutral network for phenotype *i* that create genotypes in the neutral network for phenotype *j*. Large values of this fraction indicate that phenotype *j* is relatively easy to find (via random mutations) from phenotype *i*. Mutational proximity is often not symmetric (that is, *f_ij_*≠*f_ji_*), because the denominators differ.

The first statistic estimates the overall accessibility of phenotype *i* from other phenotypes in the landscape using 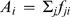. Large values of *A_i_* indicate that phenotype *i* is relatively accessible from throughout the landscape. The second statistic quantifies the potential for evolution away from phenotype *i* using a variation on Simpson's diversity index: 
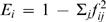
. This index indicates the diversity of other phenotypes that can be easily produced by mutations from a given phenotype, and thus may indicate the potential for further adaptation away from that phenotype. Specifically, it gives the probability that two randomly chosen *non-neutral* mutations to genotypes within a given neutral network will result in the same phenotype. The index is large for phenotypes that are adjacent to many other phenotypes, and its non-neutral mutations are fairly evenly divided among the adjacent phenotypes; it is small for phenotypes that primarily mutate to one or very few alternate phenotypes.

**Figure 4:**
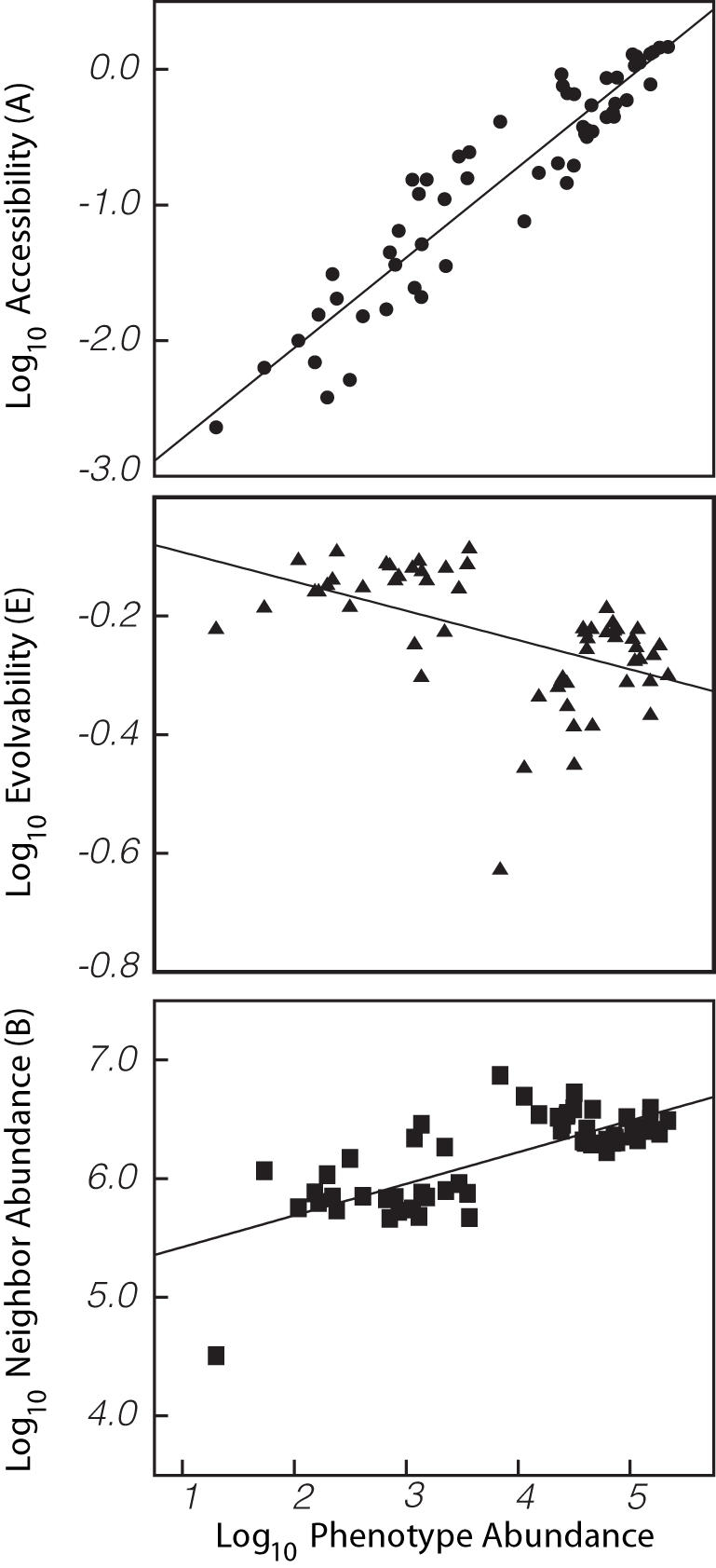
Mutational connectivity among RNA phenotypes. (Top) The Astatistic (described in text) indicates the likelihood that a given phenotype will arise through point mutation. Random mutations are more likely to hit upon larger neutral networks that smaller neutral networks (*r* ^2^ = 0.886, *P*<2.2×10^−16^; calculated on log-transformed data). (Middle) The *E* statistic (described in text) indicates the likelihood of given phenotype will produce diverse alternative phenotypes upon mutation. Point mutations to sequences in large neutral networks are less likely to yield novelty than point mutations to sequences in small neutral networks (*r* ^2^ = 0.265, *P* = 3.56×10^−5^). (Bottom) The *B* statistic (described in text) suggests that point mutations to abundant phenotypes create other abundant phenotypes (*r* ^2^ = 0.559, *P* = 1.58×10^−11^).

In the 12-mer landscape, *A* increases significantly with the abundance of a phenotype (Figure 4, top pane). In other words, random mutations are more likely to move genotypes to a large neutral network than to a small neutral network. In contrast, *E* decays significantly with phenotype abundance (Figure 4, middle pane), suggesting that it may be more difficult to evolve away from large neutral networks than small neutral networks. To provide more insight into the mutational networks, we also calculated the average abundance of phenotypes reached by mutation from phenotype *i* using 
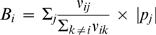
. We find that the average abundance of neighboring phenotypes significantly increases with the abundance of a phenotype (Figure 4, bottom pane), meaning that the majority of non-neutral mutations to abundant phenotypes produce other abundant phenotypes.

Thus far we have characterized the mutational networks formed by single point mutations. If we instead considered the mutational networks formed by all combinations of one, two or three mutations, then the phenotype network becomes highly interconnected. The number of adjacent phenotypes significantly increases with multiplicity of mutations considered (mean node degrees are 42.7, 53.6, and 57.2 for the one, two, and three mutant adjacencies, respectively; *P*<5×10^−3^), and the network is nearly completely connected for triple mutations. Thus, under elevated mutation rates, populations may be able to attain rare phenotypes easier than expected based on point mutation adjacencies.

In summary, these observations suggest that abundant phenotypes may be easy to find but difficult to escape, and thus the structure of a fitness landscape may significantly constrain evolutionary dynamics. Whereas the accessibility of abundant shapes is rather intuitive, the prediction that their vast neutral networks can hinder further evolution contradicts a large body of theory, which suggests that large neutral networks should enhance evolvability [18],[26],[27]. We note that this evolutionary constraint was previously proposed for a simple fitness landscape model [39].

### Mutational Networks Provide Novel Insights into Evolutionary Dynamics

To test the hypothesis that highly abundant phenotypes are readily accessible, yet poorly poised for further evolution, we ran stochastic simulations of an adapting population of 12-mer RNA molecules using an established model (see Materials and Methods for details) [18]–[21],[23]. Since we are interested in the effect of phenotype abundance on the capacity of selection to acquire the optimal phenotype, we selected the phenotypes of the founding populations (henceforth, founding phenotypes) and target shapes to span the range of abundances found among the 12-mer phenotypes. We chose ten founding phenotypes [ranks (abundance): 3 (183,791), 8 (117,213), 13 (76,478), 18 (61,699), 23 (39,740), 28 (27,312), 33 (11,354), 38 (2,260), 43 (1,299), 48 (713)] and randomly selected 20 genotypes from the neutral network of each founding phenotype to form 200 isogenic founding populations. Each founding population was composed of a single genotype and, therefore, a single phenotype. In essence, we simulated adaptation starting from 20 random points in the neutral network of each founding phenotype.

We separately adapted each founding population to twelve target phenotypes [ranks (abundance): 2 (218,576), 7 (122,332), 12 (93,866), 17 (61,895), 22 (41,092), 27 (27,522), 32 (15,348), 37 (2,963), 42 (1,368), 47 (800), 52 (240), 57 (109)]. We considered adaptation successful if the population ever acquired the target phenotype, regardless of its frequency in the population. In the successful runs, however, the target phenotype quickly dominates the populations and rises to frequencies of nearly *N* (the population size).

**Figure 5:**
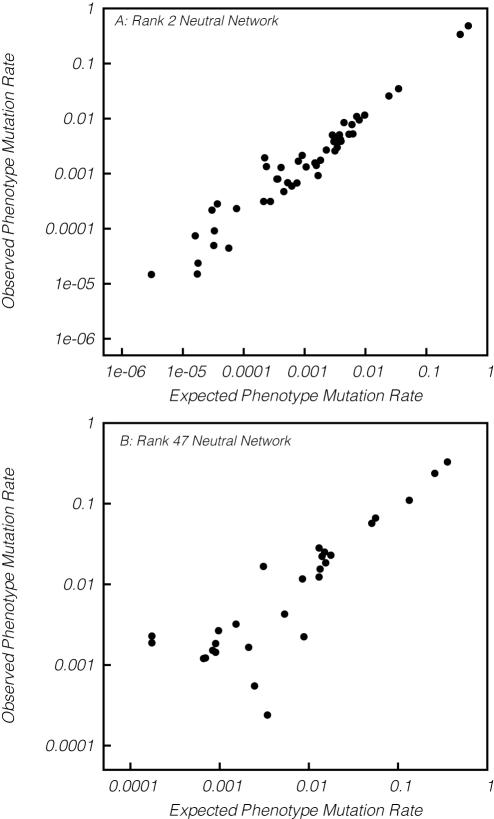
Network connectivity correlates with mutation frequency in the 12-mer fitness landscape. The rates of mutation between phenotype *i* and phenotype *j* in simulations is nearly identical to the fraction of nonneutral mutations to *i* that produce *j* (*f_ij_* ). The top pane depicts this correlation for an abundant phenotype (rank 2, 218567 sequences), whereas the bottom pane shows this for a small neutral network (rank 47, 800 sequences). The mean slope of the regression line (taken over all 52 of 59 neutral networks that arose in simulation) was *r* ^2^ = 0.978 with 95% confidence interval [0.945, 1.011], which is statistically indistinguishable from one.

The mutational connectivity statistics described above (*A_i_* and *E_i_*) will only be good indicators of evolutionary dynamics if the probability of mutating from phenotype *i* to phenotype *j* correlates with the fraction of mutations to *i* that produce *j* (*f_ij_*). To test this basic assumption, we compared the phenotype mutation rates observed in the simulations (fraction of mutations to *i* that produce *j*) to *f_ij_* (the fraction of non-neutral point mutations to genotypes in the neutral network for phenotype *i* that create genotypes in the neutral network for phenotype *j*). In fact, we find an almost perfect relationship between the two quantities (Figure 5A), suggesting that mutational network structure fundamentally constrains evolution and that *A_i_* and *E_i_* are good indicators of these constraints.

**Figure 6:**
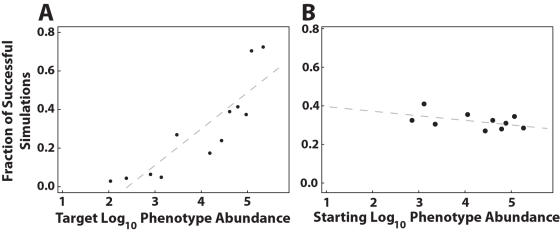
Stochastic evolutionary simulation in the 12-mer fitness landscape. (A) The phenotype abundance of the target strongly affects the success of adaptation (*r* = 0.76, *P* = 2.2×10^−4^). (B) The phenotype abundance at the start of the simulation has no effect on the evolutionary outcome (*r* = −0.023, *P* = 0.17). We simulated adaptation over one million generations with a genomic mutation rate of *U* = 0.0003 and a constant population size of N = 1000.

**Figure 7:**
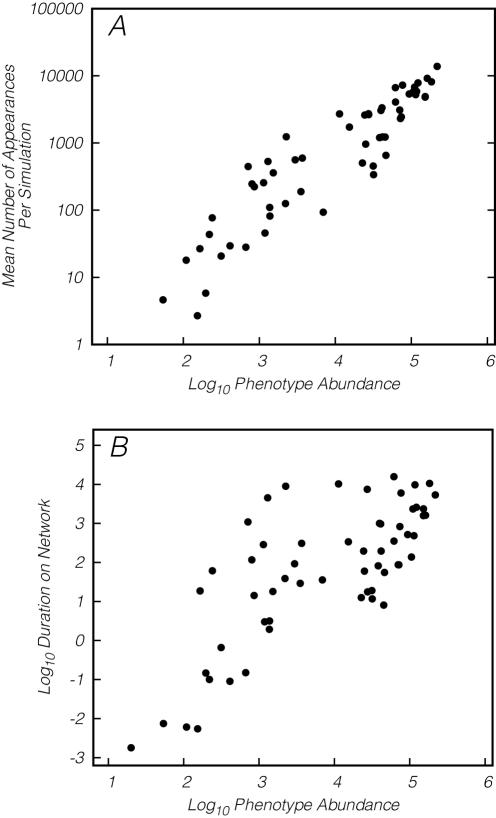
Populations exploring the 12-mer fitness landscape. (A) The number of appearances of a phenotype is strongly correlated with the abundance of that phenotype (*r* = 0.92, *P* = 2.2×10^−16^, calculated on logtransformed data). (B) The total number of time steps that a phenotype occurs in the evolving populations is positively correlated with its abundance (*r* = 0.75, *P* = 1.5×10^−11^, calculated on logtransformed data).

Across the 2400 simulations, we observed a significant positive correlation between the abundance of the target phenotype and the likelihood that a population successfully evolved to the target (Figure 6A). This is consistent with the positive relationship between phenotype abundance and mutational accessibility, as indicated by the A statistic (Figure 4A). Phenotype abundance also positively correlates with the number of times a phenotype arises in the evolving populations (Figure 7A). Taken together, these results support our hypothesis that abundant shapes are more likely to appear via mutation in evolving populations than are rare shapes.

We did not, however, observe a relationship between the founding phenotype abundance and the ultimate evolutionary outcome (Figure 6B). When a simulation failed to acquire the target, the population was primarily composed of phenotypes of greater abundance than both the target phenotype and the average abundance of a random phenotype, demonstrating that the structure of mutational networks can steer populations towards abundant, but non-optimal, phenotypes. As suggested by the negative relationship between abundance and the *E* statistic, evolution away from abundant phenotypes appears to be limited by the improbability of beneficial mutations. In support of this explanation, we also find a significant positive correlation between the abundance of a phenotype and the duration of the phenotype in the evolving populations (Figure 7B).

These observations appear to be inconsistent with the widely-held belief that neutral networks facilitate evolution by allowing populations to traverse large regions of fitness landscapes without reducing fitness [15], [18]–[20],[26],[27],[40]. In our simulations, populations readily evolve from one abundant shape to another (that is, from one large neutral network to another), but are often unable to evolve rare phenotypes. Thus, while the hypothesis that neutrality (the fraction of mutations that are neutral) allows populations to explore phenotype space is true, the evolutionary outcome of such exploration is generally confined to other abundant phenotypes. Most of the prior studies addressing this hypothesis are based on relatively small random samples of sequences from large genotype spaces, which may consist of exclusively abundant phenotypes. The conclusion that neutrality facilitates evolution is reasonable when considering only abundant subsets of fitness landscapes, but is somewhat misleading when one considers the fitness landscapes in their entirety.

### The “Ascent of the Abundant” and the Evolution of Natural RNA Molecules

These results suggest the following hypothesis: the evolution of phenotypes, whether complex whole-organism phenotypes or RNA shapes, may be biased toward abundant phenotypes, even if those phenotypes are not optimal. We cannot, however, test this hypothesis by directly measuring the abundances of complex organism-level phenotypes since we cannot yet completely characterize their fitness landscapes. As a first step in this direction, we have developed a simple structural statistic that allows us to indirectly estimate the abundances of naturally occurring RNA shapes, which are much larger and more complex than those considered thus far.

**Figure 8:**
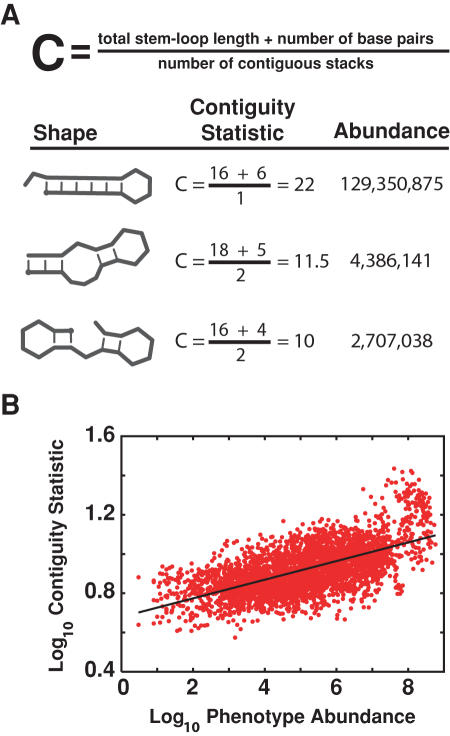
Calculation of the contiguity statistic. (A) Sample calculations of the contiguity statistic for three 18-mer shapes. (B) The contiguity statistic is strongly correlated with abundance for all lengths of RNA molecules studied; example shown is the 18-mer land-scape (*r* ^2^ = 0.69, *P*<2.2×10^−16^). Minimal Gaussian noise was added to reduce granularity in the data.

Across the *n*-mer phenotypes, we observed that longer contiguous helical stacks (stems) form more frequently than shorter contiguous stacks and stacks that contain bulges (which break up helices). We quantify this with a new statistic (Figure 8) given by
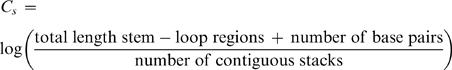



This *contiguity statistic* significantly correlates with log phenotype abundance in the 12- through 18-mer landscapes [*r* ranges from *r* = 0.71 (*P* = 3.6×10^−10^) in the 12-mer landscape to *r* = 0.69 (*P*<2.2×10^−16^) in the 18-mer landscape]. The utility of the contiguity statistic is that one genotype is sufficient to estimate the abundance of its phenotype. We conjecture, therefore, that we can use the contiguity statistic to ask whether naturally occurring RNA molecules are biased towards abundant shapes.

We used the contiguity statistic to estimate the abundances of the RNA molecules in Rfam, a curated database of functional RNA genes [36]. The Rfam molecules are grouped into families, and every sequence in a family is thought to code for the same functional RNA. We compared the contiguity statistics calculated for the Rfam sequences to null distributions generated by calculating contiguity statistics for thousands of random permutations of those sequences. Specifically, for each naturally evolved molecule, we determined whether the contiguity statistics of their predicted shapes were significantly larger than the contiguity statistics of random molecules from the same fitness landscape (see Methods for details).

The structures of the natural RNA molecules indeed have larger contiguity statistics than randomly chosen structures from the same fitness landscapes (Figure 1). This observation supports an “ascent of the abundant” hypothesis in which the mutational networks connecting diverse phenotypes may steer populations toward abundant, though not necessarily optimal, phenotypes. Yet, Figure 1 (red squares) shows that natural molecules are also significantly more thermostable than random molecules. Thus one must ask whether the high contiguity values of natural molecules are simply byproducts of the evolution of thermostability (or some other advantageous structural property) or, in fact, exist because of mutational biases towards abundant shapes, or both.

The abundances of the natural molecules (as estimated by their contiguity statistics) are even more statistically pronounced than their thermostabilities. We used logistic regression analysis to ask which of contiguity or thermostability better distinguishes naturally occurring molecules from their random permutations. We regressed molecule class (natural or random permutation) on contiguity statistic and (separately) on thermostability. The area under a receiver operating curve (ROC) gives the probability that a model correctly assigns a binary variable (natural or random molecule) to its proper group. The logistic model for contiguity yielded an area under the ROC of 0.82, which is good; the model for thermodynamic stability yielded an area under the ROC of 0.62, which is poor. Our results are therefore consistent with an apparent biases towards abundant phenotypes in both the small RNA landscapes and natural RNAs are not simply byproducts of natural selection for thermostability.

## Discussion

Evolutionary biologists have long appreciated that the evolutionary potential of a phenotype depends on the breadth of its neutral network. Eigen's error catastrophe theory, an extension of classic mutation-selection balance theory, argues that the evolutionary potential of a phenotype depends on both its fitness relative to alternative phenotypes and its robustness to mutations [41]. Under high mutation rates, only phenotypes with sufficiently large and connected neutral networks can persist. The phrase “survival of flattest” has been used to refer to the evolutionary success of low-fitness phenotypes with large neutral networks over higher-fitness phenotypes with small neutral networks [42]. Critically, this idea assumes that these diverse phenotypes compete directly with one another in an evolving population.

The relationship between abundance and evolvability that we have described here is not a simple restatement of this idea. Instead, the evolutionary tendency towards abundant phenotypes results from a biased exploration of phenotype space. Abundant phenotypes are more discoverable (random mutations are more likely to produce abundant phenotypes) and more inescapable (once abundant phenotypes evolve, it is very hard to mutate to other phenotypes). In our simulations, we observed that, when the populations failed to acquire the target phenotype, it was not due to the target shape being lost to mutation pressure or other forces. In the failed simulations, the target phenotype never appeared in the first place (not shown).

Our results extend ideas developed in prior studies of both RNA and protein structural evolution [15],[29]. In particular, Schuster et al. argued that abundant RNA phenotypes are within a few mutations of almost any genotype in the landscape [15], and Reidys et al. further demonstrated that only abundant phenotypes have neutral networks that percolate through the entire sequence space [24]. As a result, evolutionary biologists have proposed that large neutral networks greatly enhance the evolutionary potential of evolving populations [15],[18],[24],[26],[27]. Yet, these studies largely focused on the local structure of neutral networks and not global patterns of mutational connectivity.

Here we have taken a global perspective and found that large neutral networks are more likely to impede than enable evolution. The probability of a non-neutral mutation and the diversity of phenotypes produced by such mutations both decline as neutral network size increases (Figure 4, middle). In our simulations, populations on large neutral networks were no more likely to evolve better phenotypes than populations on small neutral networks (Figure 6). Furthermore, these populations spent more time on large neutral networks than small neutral networks (Figure 7B).

Our results more generally suggest that the structure of RNA mutational networks favors the evolution of abundant phenotypes, even when rare phenotypes are more fit. Abundant phenotypes are more likely to arise via a random mutation than rare phenotypes, and, once established in the population, are more difficult to escape via subsequent mutations. This gives a new perspective on the widely-accepted hypothesis that large neutral networks facilitate evolution [15],[18],[24],[26],[27]. While large neutral networks enable populations to *explore* large regions of fitness landscapes via mutation, the outcome of such exploration is almost always evolution to another abundant phenotype rather than to a rare phenotype. Thus, in the larger scheme of things, neutrality may serve as a trap rather than a catalyst for evolution.

While our study suggests that naturally occurring RNA molecules are biased towards abundant shapes, we recognize that abundance may have evolved as a byproduct of correlated biophysical or biochemical properties that enhance the functionality of molecules. We specifically address the possibility that the abundance bias may be driven by thermostability. Our simulation study shows that abundant shapes will evolve in the absence of natural selection for thermostability, and our analysis of natural RNA molecules indirectly suggests that thermostability alone cannot account for the bias toward abundant shapes. We believe that both processes have probably contributed to the prevalence of abundant shapes: (i) natural selection for thermostability and/or other beneficial molecular properties that correlate with abundance and (ii) the underlying structure of the mutational network. We contend that the second process is important and perhaps has precluded the evolution of functionally optimal molecules.

In closing, we have further characterized the relationship between phenotype abundance and mutational connectivity, and explored its evolutionary implications. The abundance of a phenotype positively correlates with the probability of randomly mutating to that phenotype and negatively correlates with the probability of randomly mutating away from that phenotype to alternative phenotypes. Consequently, the evolutionary potential of a phenotype critically depends on its abundance, and mutational networks therefore can fundamentally constrain evolution. As we learn more about the structure of mutational networks, we can gain new perspectives on the history and function of natural systems and better methods for artificially selecting molecules with desired functions. Characterizing mutational networks remains a formidable challenge, particularly when we consider more complex phenotypes and sources of variation beyond simple point mutations. We can approach these larger landscapes using statistical shortcuts, like the contiguity statistic introduced here, that indirectly provide information about the global structure of the fitness landscape, or by designing farther-reaching mutagenesis experiments.
